# Effect of ANA positivity on clinical picture of the JIA: should ANA positive JIA be classified as a different group?

**DOI:** 10.1186/1546-0096-9-S1-P156

**Published:** 2011-09-14

**Authors:** Adem Polat, Erkan Demirkaya, Turker Turker, Yelda Bilginer, Erbil Unsal, Muferet Erguven, Hakan Poyrazoglu, Ozgur Kasapcopur, Faysal Gok, Seza Ozen, for the FMF Arthritis Vasculitis and Orphan Disease Research in Paediatric Rheumatology (FAVOR)

**Affiliations:** 1 Gülhane Military Medical Academy, Pediatric Nephrology & Rheumatology Unit, Ankara, Turkey; 2 Gulhane Military Medical Faculty, Department of Public Health, Division of Epidemiology, Ankara, Turkey; 3 Hacettepe University Medical School, Pediatric Nephrology & Rheumatology Unit, Ankara, Turkey; 4 Dokuz Eylul University Medical School, Pediatric Immunology and Rheumatology Unit, İzmir, Turkey; 5 Goztepe Training and Research Hospital, Division of Pediatric Rheumatology, Istanbul, Turkey; 6 Erciyes University Medical School, Pediatric Nephrology & Rheumatology Unit, Kayseri, Turkey; 7 Istanbul University Cerrahpasa Medical School Department of Pediatric Rheumatology, Istanbul, Turkey

## Background

According to ILAR classification, JIA is classified into 7 different categories based on similar characteristic features of each.

## Aim

In this study we aimed to investigate whether ANA positive patients in different ILAR categories constitute a homogenous group.

## Methods

In this cross-sectional study, patients who had been followed up for at least a 6 month-period were recruited from different centers and registered thorough a web-based registry. Patients were grouped according to their ANA positivity. Clinical and demographic features were compared between ANA positive and negative groups. The results were explored by univariate and multivariate regression analysis (OR, %95 CI).

## Results

A total number of 402 JIA patients of which 169 ANA positive and 233 ANA negative were enrolled in the study. The mean age of the diagnosis in ANA negative and positive groups were 4.65 ± 3.48 and 4.01 ± 2.86 respectively; and female-male ratio for ANA negative and positive groups were 1.3 (135/98) and 4.1 (136/33) respectively. The subgroups of the patients according to ILAR classification system are listed in the table.

According to our results of multipl regression analysis, the variables which demonstrated statisticly significant association with ANA presence were female sex (OR =3.763 (2.26-6.27)), uveitis (OR=5.58 (2.21-14.10)), rheumatoid factor positivity (OR=4.07 (1.34-12.36)), high levels of ESR (OR 0.993 (0.985-1.00)) and small joint involvement (OR 0.57 (0.31-1.03)).

## Conclusion

Our results indicate that, ANA positive patients classified in different groups by using current ILAR classification system, demonstrate similar clinical and laboratory findings. Thus we suggest that ANA status may be used as a parameter for classification of JIA.

**Table 1:** 

	ANA negative		ANA pozitive		P
		
	n	%	n	%	
Oligoarticular persistent	113	48,5	121	71,6	<0,001
Oligoarticular extended	11	4,7	15	8,9	
Polyarticular RF (-)	99	42,5	30	17,8	
Psoriatic	10	4,3	3	1,8	
Total	233	100	169	100	

